# Ras2 Is Responsible for the Environmental Responses, Melanin Metabolism, and Virulence of *Botrytis cinerea*

**DOI:** 10.3390/jof9040432

**Published:** 2023-03-31

**Authors:** Hua Li, Xuemei Shen, Wenjia Wu, Wanyu Zhang, Yousheng Wang

**Affiliations:** 1 School of Light Industry, Beijing Technology and Business University, Beijing 100048, China; 2 Rizhao Huawei Institute of Comprehensive Health Industries, Shandong Keepfit Biotech. Co., Ltd., Rizhao 276800, China

**Keywords:** *Botrytis cinerea*, Ras, melanin, environmental response, virulence

## Abstract

Ras proteins are monomeric G proteins that are ubiquitous in fungal cells and play important roles in fungal growth, virulence, and environmental responses. *Botrytis cinerea* is a phytopathogenic fungus that infects various crops. However, under specific environmental conditions, the overripe grapes infected by *B. cinerea* can be used to brew valuable noble rot wine. As a Ras protein, the role of Bcras2 in the environmental responses of *B. cinerea* is poorly understood. In this study, we deleted the *Bcras2* gene using homologous recombination and examined its functions. Downstream genes regulated by *Bcras2* were explored using RNA sequencing transcriptomics. It was found that Δ*Bcras2* deletion mutants showed significantly reduced growth rate, increased sclerotia production, decreased resistance to oxidative stress, and enhanced resistance to cell wall stress. Additionally, *Bcras2* deletion promoted the expression of melanin-related genes in sclerotia and decreased the expression of melanin-related genes in conidia. The above results indicate that *Bcras2* positively regulates growth, oxidative stress resistance, and conidial melanin-related genes expression, and negatively regulates sclerotia production, cell wall stress resistance and sclerotial melanin-related genes expression. These results revealed previously unknown functions of *Bcras2* in environmental responses and melanin metabolism in *B. cinerea*.

## 1. Introduction

*Botrytis cinerea* is an aggressive pathogenic fungus that infects more than 1000 plant species, particularly fresh fruits and vegetables, resulting in economic losses of up to $10 to $100 billion annually worldwide [1]. Almost all plant tissues can be infected by *B. cinerea*, including flowers and fruits [2,3]. Whether fruit rot occurs after infection depends on the mature state of the fruit and the environmental conditions [4]. *B. cinerea* tends to remain quiescent on immature fruits and causes gray mold disease in ripe or senescent fruits under conducive environmental conditions [5]. Interestingly, under special environmental conditions such as cold humid nights and sunny dry days, *B. cinerea*-infected overripe grapes will not rot rapidly but become botrytized grapes (noble rot) [6]. Botrytized grapes have a shriveling berry and an increased concentration of glucose, acids, and other metabolites due to water evaporation caused by grape skin cracks made by *B. cinerea* [7]. Wines made from botrytized grapes, called botrytized wine, have an attractive taste and numerous health benefits for human beings [8]. Therefore, environmental conditions play an important role in the pathogenicity of *B. cinerea*. Revealing the response mechanism of *B. cinerea* to external environmental conditions contributes to the development of new strategies for controlling gray mold disease, as well as new technologies for producing botrytized grapes.

Ras proteins are prototypical members of the small monomeric GTPase superfamily that play key roles in regulating cellular responses to various environmental stresses by activating a wide range of downstream signaling effectors [9]. Although there are many Ras members in humans, most filamentous fungi contain only 2–3 Ras proteins with similar or distinct functions [10]. As a signal transducer, Ras has a guanosine nucleotide diphosphate (GDP) or guanosine nucleotide triphosphate (GTP) binding status. The activity control of Ras is mediated by switching between active GTP and inactive GDP-binding status [11]. Proper cellular localization of Ras proteins within the cell is also important for their function [12]. In *Sporisorium scitamineum,* two Ras proteins with similar roles were identified. Ras1 is responsible for normal growth, mycelial morphology, sensitivity to cell wall stressors, and altered chitin content. Ras2 deletion mutants exhibit notably slower growth, shorter and thicker mycelia, weakened sexual mating capacity, and hypersensitivity to cell wall stressors. The normal function of Ras requires a cell membrane location; a strain with Ras dislocation shows weak sexual mating ability and strong sensitivity to cell wall stresses [13]. In *Beauveria bassiana*, Ras1 and Ras2 have different roles. Ras1 was down-regulated under hyperosmotic stress, whereas Ras2 was up-regulated. Ras1 is responsible for asexual development, as constitutively active (Ras1G19V) and inactive (Ras1D126A) mutants showed different colony sizes, hyphal morphology, and conidiogenesis. The Ras2 mutant also showed altered hyphal morphology and multiple germ tubes, but Ras1G19V expression in the Ras2 mutant did not complement the multiple germ tube defects [14]. Besides Ras1 and Ras2, a third Ras protein (Ras3) in *B. bassiana* was found, which functions upstream of the Hogl signaling pathway and is required for conidiation, multi-stress tolerance, and virulence [15]. In *B. cinerea*, there are two Ras proteins with different roles. Ras1 deletion strains had severe growth defects: hyperbranched and deformed hyphae. In addition, the mutants are vegetatively sterile and completely non-pathogenic. A close cross-talk between BcRas1 and BcSAK1 MAPK pathways may exist because the phenotypes of BcRas1 and BcSAK1 deletions were basically consistent [16]. Although Ras2 has been reported to affect germination and virulence [17], its function in the environmental stress response and the downstream signaling pathway requires further study.

In this study, we analyzed the function of Ras2 in growth, development, cell stress responses, virulence, and its possible downstream targets. Our results showed that Δ*Bcras2* had lower growth rates, reduced virulence in apple fruit and green peppers, decreased resistance to oxidative stress, and enhanced resistance to cell wall stress. Further RNA sequencing analysis showed that various membrane-related proteins and a key sclerotial melanin-related gene were overexpressed, and several conidial melanin-related genes was repressed in Δ*Bcras2*. These results revealed previously unknown functions of *Bcras2* in environmental responses and melanin metabolism in *B. cinerea*. 

## 2. Materials and Methods

### 2.1. Fungal Strains and Culture Conditions

*B. cinerea* B05.10 was used as the wild-type control and the recipient strain in the transformation experiments to generate gene deletion mutants. *B. cinerea* strains were cultured on complete medium (CM) or potato dextrose agar (PDA) plates at 25 °C. 

### 2.2. Generation of Gene Deletion Mutants

Deletion mutants were constructed using the homologous recombination strategy. Approximately 1 kb regions immediately upstream of the initiation codon and downstream of the termination codon were amplified and cloned into the pLOB7 vector to flank the hygromycin resistance box to form the replacement vector. The replacement vector was confirmed by sequencing and amplified using a high-fidelity enzyme. Amplification products were purified and directly introduced into protoplasts of the wild-type. Protocols for protoplast formation and transformation were performed according to a previous study [18]. Briefly, mycelia of *B. cinerea* were treated with Glucanex solution (0.5%, Sigma-Aldrich, St. Louis, MI, USA) at 25 °C for 2–3 h to produce protoplasts. Fragments for conversion were added to 200 μL protoplasts. Thereafter, 25% polyethylene glycol (PEG) 3350 dissolved in 50 mM CaCl_2_ and 10 mM Tris-HCl (pH 7.5) were added into the protoplasts. The mixture was evenly distributed on six SH plates (0.6 M sucrose, 5 mM HEPES, 1 mM (NH4)_2_HPO4, and 1% agar). SH medium containing hygromycin B was placed on the surface of SH plates to screen the transformants. Diagnostic PCR was performed to verify integration of the selected transformants.

To purify the homokaryotic deletion mutants, spores of the mutants were collected with sterile distilled water and counted using a hemocytometer after 10 days of culture on PDA medium. The strains were then transferred onto PDA plates for DNA isolation. Primers outside the hygromycin resistance region were used to detect the homokaryon. Primer information is shown in Table S1. Only one DNA band could be amplified from the homokaryon, with a size inconsistent with that of the wild-type.

Southern blotting was performed to rule out ectopic recombination. The genomic DNA of the deletion mutants and the wild-type were extracted and digested with restriction enzymes. The hygromycin resistance region was used as a probe by amplifying with digoxigenin-dUTP. Southern blot experiments were performed as previously described [19]. Primer information is shown in Table S1.

### 2.3. Phenotype Analysis

For growth assays, conidia of Δ*Bcras2* mutants and the wild-type were harvested with sterile distilled water, and the conidial suspension was adjusted to 5 × 10^5^ conidia mL^−1^. A conidial suspension (5 μL) was added to the CM or PDA plates to observe mycelial growth. The colony diameter was measured using the crossing method. 

For stress response analysis, conidial suspension (5 × 10^5^ conidia mL^−1^) of Δ*Bcras2* mutants and the wild-type were dropped into the center of CM plates supplemented with the osmotic stress agents KCl (0.5 M, 1 M) and NaCl (0.5 M, 1 M), the oxidative stress agent H_2_O_2_ (3 mM, 6 mM), cell wall disturbing agents Congo Red (CR, 150 μg mL^−1^, 300 μg mL^−1^), and sodium dodecyl sulphate (SDS, 0.005%, 0.01%). The colony diameter was determined and the relative mycelial growth inhibition rate (RMGI, c = [(C − N)/(C − I)] ×100%) was calculated according to a previous study [20]. C refers to the diameter of the colony cultured under no pressure, N refers to the diameter of colony cultured under the simulated stress conditions, and I refers to the diameter of the inoculated mycelial plugs. All experiments were repeated at least three times.

### 2.4. Virulence Assays

The Δ*Bcras2* mutants and the wild-type strain were transferred several times on CM plates to ensure consistent growth conditions. Apple fruit or green peppers of similar size and maturity were chosen, surface-disinfected with 2% (*v*/*v*) sodium hypochlorite solution for 2–3 min, and wounded to the same depth (2 mm wide and 3 mm deep) using a sterile nail. Mycelial plugs (3 mm in diameter) or conidial suspension (5 × 10^5^ conidia mL^−1^) of Δ*Bcras2* mutants and the wild-type were inoculated into the apple or green pepper wounds, respectively. Lesions were measured 3–5 days after inoculation. The experiment was repeated three times. 

### 2.5. RNA Sequencing 

Mycelia for three independent biological replicates were collected and put into 1.5 mL centrifuge tubes, frozen in liquid nitrogen, and stored at −80 °C for RNA extraction. Total RNA from mycelia was extracted using TRIzol, and its quality was detected using Nanodrop2000, Agarose gel electrophoresis, and 2100 Bioanalyzer (Agilent Technologies, Santa Clara, CA, USA). The RNA from two biological replicates was carefully purified and the cDNA library was constructed by Majorbio (Shanghai, China). The mRNA was isolated from total RNA using magnetic beads with Oligo (dT); sequencing was performed using an Illumina NovaSeq 6000. The paired-end reads were 150 bp in size. Raw data were filtered to obtain the clean (high-quality) data for subsequent analyses. The ratio of Q30 and GC content of the clean data were calculated. Clean data were aligned to the reference genome (http://fungi.ensembl.org/Botrytis_cinerea/Info/Index, accessed on 9 September 2022), and the data were evaluated again by a homogeneity distribution check and sequencing coverage situation analysis. HISAT2 (https://daehwankimlab.github.io/hisat2, accessed on 9 September 2022) software was used for sequence alignment analysis [21]. To ensure the reliability of biological duplication, correlation analysis and principal component analysis (PCA) were carried out. Gene expression level was estimated by TPM (Transcripts Per Million reads). The DESeq2 software was used to analyze differential gene expression [22]. Genes with *p*-adjust < 0.05 and FC (fold change) ≥2 or ≤0.5 were regarded as significantly different expressions. 

The GO, KEGG, COG, NR, Swiss-Prot, and Pfam database was used for functional annotations. The software Goatools (https://github.com/tanghaibao/Goatools, accessed on 9 September 2022) was used to conduct GO enrichment analysis on the transcripts in the gene set [23]. Benjamini-Hochberg (BH) multiple testing correction was used for the adjusted *p*-values. GO function with corrected *p*-values (FDR) less than 0.05 was considered to be significantly enriched. 

Co-expression was analyzed using software RESM with Pearson correlation algorithm. Benjamini-Hochberg (BH) multiple testing correction was used for the adjusted *p*-values. Genes pairs with correlation coefficient greater than 0.99 and adjusted *p*-values less than 0.05 were selected. Cytoscape software was used to draw co-expression networks [23].

### 2.6. Gene Expression Analysis

The method of mycelia culture was the same as for RNA sequencing. Total RNA from mycelia was isolated using an RNA extraction reagent (Trizol, Thermo Fisher, Waltham, MA, USA). First-strand cDNA for RT-qPCR was prepared using PrimeScript™ RT reagent Kit with gDNA Eraser (Perfect Real Time) (RR047A, Takara, Shiga, Japan) from one 1 µg of total RNA, and TB Green Premix Ex Taq II (Tli RNaseH Plus) (RR820A, Takara) was used to perform PCR. The relative expression levels were calculated as previously described. Ubiquitin-conjugating enzyme (UCE) was used as the reference gene [24], and the primers used are shown in Table S1. The experiment was repeated three times. 

### 2.7. Statistical Analysis

Statistical analysis was performed using Microsoft Excel and GraphPad Prism 6. All data were expressed as the mean ± standard deviation (SD) (*n* ≥ 3). The least significant difference (LSD) test and one-way analysis of variance (ANOVA) were used to examine the significant differences among samples. *, **, *** indicate significance at *p* < 0.05, *p* < 0.01, and *p* < 0.001, respectively. 

## 3. Results

### 3.1. Bcras2 Is Required for Mycelial Growth and Development

**Figure 1:**
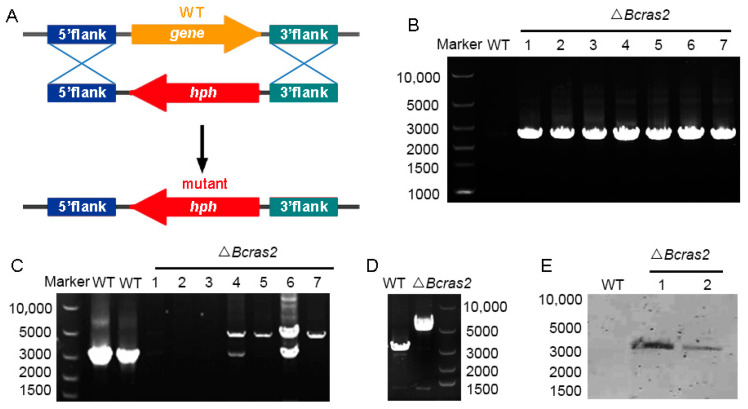
Generation of Δ*Bcras2* mutants: (**A**) Strategy for constructing the Δ*Bcras2 deletion mutants*. hph, the hygromycin resistance cassette. (**B**) PCR diagnosis of Δ*Bcras2* mutants. (**C**,**D**) Diagnosis of the homozygotes of Δ*Bcras2* mutants. The picture showed the results from two PCR tests. (**E**) Southern blot analysis of the wild-type (WT) and Δ*Bcras2* strains. Numbers represent different strains.

Deletion mutants of *Bcras2* (Δ*Bcras2*) were generated by replacing the open reading frame (ORF) of *Bcras2* with a hygromycin resistance cassette (hph) in wild-type *B. cinerea* strain B05.10 (Figure 1A). At least seven deletion mutants were obtained, as shown by the diagnostic PCR (Figure 1B). Three homokaryotic strains were gained by several rounds of single-spore isolation (Figure 1C,D) and named Δ*Bcras2-1*, Δ*Bcras2-2*, Δ*Bcras2-3* thereafter. We further performed Southern blot detection on two homokaryotic mutants. Southern blot analysis revealed that there were no ectopic integrations (Figure 1E). 

**Figure 2:**
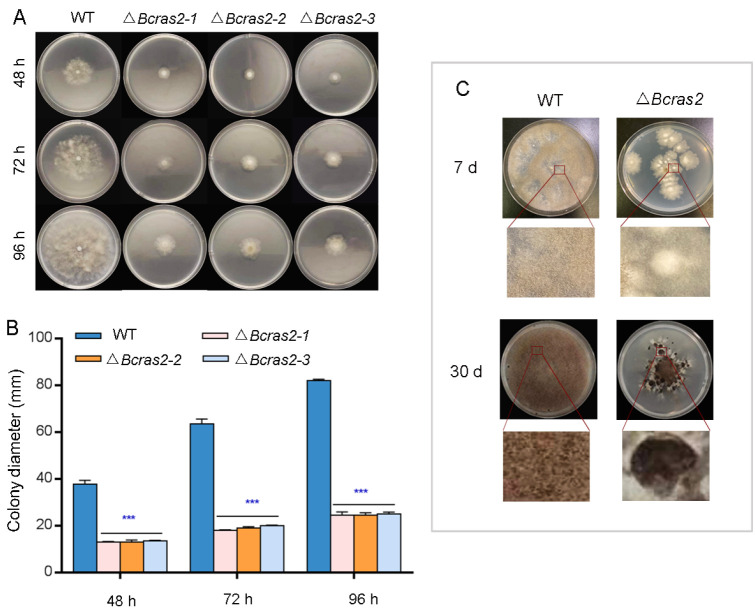
Phenotype detection of Δ*Bcras2* mutants: (**A**) Colony morphology of Δ*Bcras2* mutants and the wild-type on complete medium (CM) plates. (**B**) Statistical analysis of colony diameter. (**C**) Colony morphology of Δ*Bcras2* mutants and the wild-type on potato dextrose agar (PDA) plates. Strains were cultured at 25 °C. Phenotype detection experiments were repeated three times with similar results, and the pictures shown are representative. Bars represent standard deviations (SD) of the means from three CM plates. *** indicate significant differences (*p* < 0.001).

To determine the role of *Bcras2* in the growth of *B. cinerea*, we compared the radial growth rates and morphogenetic phenotypes of B05.10 and three Δ*Bcras2* mutants on complete medium (CM). As shown in Figure 2A, three Δ*Bcras2* mutants all showed reduced growth rates and altered colony morphology. The radial growth rates were significantly reduced to 35%, 30%, and 30% at 48 h, 72 h, and 96 h, respectively (Figure 2B). The hyphae of the wild-type were outstretched, whereas those of the Δ*Bcras2* mutants were smaller and denser. 

The development of Δ*bcras2* mutants and the wild-type was detected on potato dextrose agar (PDA) plates. After 7 days of culture, the colonies of the Δ*bcras2* mutants were significantly smaller and slightly whiter than those of the wild-type. Compared with the wild-type, less conidia were generated by Δ*bcras2* mutants. On the 30th day, the center of Δ*bcras2* colonies darkened since the hyphae were compact and a large number of conidia gathered at the center. A circle of black sclerotia appeared around the colonies of the Δ*bcras2* mutants. Compared to the wild-type, Δ*bcras2* mutants had more, bigger, and darker sclerotia (Figure 2C).

### 3.2. Bcras2 Is Responsible for Oxidative Stress Responses and Cell Wall Integrity

**Figure 3:**
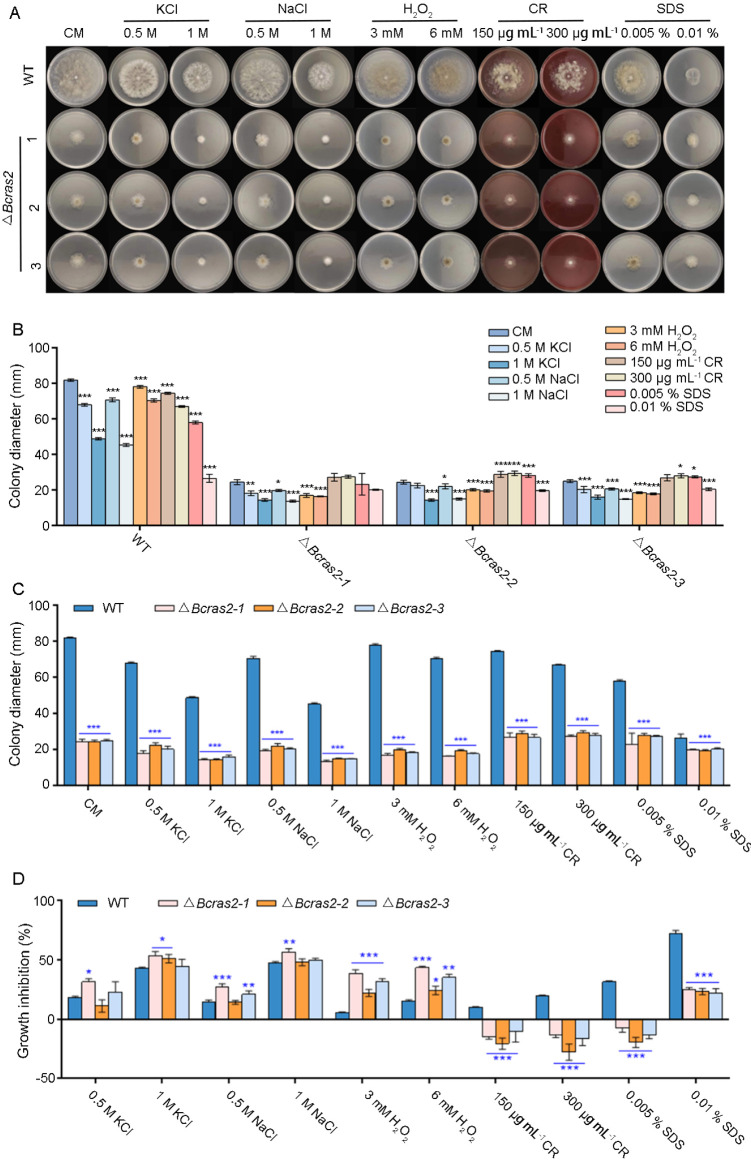
*Bcras2* mediates oxidative stress response and cell wall integrity of *B. cinerea*. (**A**) Conidial suspension (5 × 10^5^ conidia mL^−1^) of Δ*Bcras2* mutants and the wild-type were dropped into CM plates supplemented with the osmotic stress agents KCl (0.5 M, 1 M) and NaCl (0.5 M, 1 M), the oxidative stress agent H_2_O_2_ (3 mM, 6 mM), cell wall disturbing agents Congo Red (CR, 150 μg mL^−1^, 300 μg mL^−1^), and sodium dodecyl sulphate (SDS, 0.005%, 0.01%). (**B**,**C**) Quantification of the relative hyphal growth of different strains on plates with/without stress agents. (**D**) The relative hyphal growth inhibition of the strains on plates supplemented with/without stress agents. Representative photos were taken 3 days post-inoculation. *, **, *** significance at *p* < 0.05, *p* < 0.01, and *p* < 0.001, respectively.

To reveal the role of *Bcras2* in the environmental stress responses of *B. cinerea*, the growth rates of Δ*Bcras2* mutants and the wild-type were compared on complete medium containing stressors. Osmotic stress agents KCl (0.5 M, 1 M) and NaCl (0.5 M, 1 M), the oxidative stress agent H_2_O_2_ (3 mM, 6 mM), and the cell wall disturbing agents Congo Red (CR, 150 μg mL^−1^, 300 μg mL^−1^) and sodium dodecyl sulfate (SDS, 0.005%, 0.01%) were used (Figure 3A). 

The growth of wild-type *B. cinerea* was inhibited by stressors in a dose-dependent manner. Osmotic stress agents KCl and NaCl, and the cell wall disturbing agent SDS had more obvious inhibitory effects on growth than other stressors. The inhibitory effect of osmotic stress agents KCl and NaCl on the three Δ*Bcras2* mutants was similar to that of wild type. However, cell wall disturbing agents CR (150 μg mL^−1^, 300 μg mL^−1^) and SDS (0.005%) were beneficial to the growth of the mutants (Figure 3B).

All the Δ*Bcras2* mutants showed significantly reduced growth when compared with the wild-type on CM plates (Figure 3C). The addition of different agents has diverse effects on growth defects. In the osmotic stress test, the growth inhibition of the Δ*Bcras2*-1 mutant was significant, whereas that of other mutants was not. As for oxidative stress, all mutants showed a greater growth defect than the wild-type. However, the growth defect of the Δ*Bcras2* mutants was attenuated by cell-disrupting agents such as CR and SDS. All Δ*Bcras2* mutants grew better on CR-containing CM plates than on CM plates in a dose-dependent manner. Interestingly, although Δ*Bcras2* mutants also grew better on SDS-containing plates (0.005 %) than on CM plates, the growth of Δ*Bcras2* mutants was inhibited at a higher concentration (0.01%) (Figure 3D). 

### 3.3. Bcras2 Is Essential for Virulence

**Figure 4:**
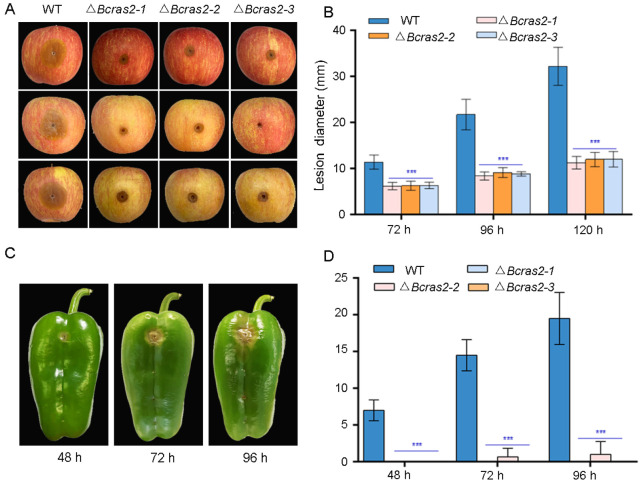
Virulence assays of Δ*Bcras2* mutants and the wild-type on apples and green peppers. (**A**) Disease symptoms of Δ*Bcras2* mutants and the wild-type on apple fruit. Images were captured 96 h post-inoculation. (**B**) Statistical analysis of Δ*Bcras2* mutants and the wild-type on apple fruit. *** indicate significant differences (*p* < 0.001). (**C**) Disease symptoms of Δ*Bcras2* mutants and the wild-type on green peppers. Images were captured 48 h, 72 h, and 96 h post-inoculation. (**D**) Statistical analysis of Δ*Bcras2* mutants and the wild-type on green peppers. *** indicate significant differences (*p* < 0.001).

To investigate the role of *Bcras2* in *B. cinerea* virulence in planta, we inoculated apple (*Malus pumila* Mill.) fruit and green peppers (*Capsicum annuum* L.) with mycelial plugs and conidia, respectively. Δ*Bcras2* mutants showed reduced virulence in apples (Figure 4A). Obvious lesions were observed 72 h after the inoculation of apple fruit wounds, in which the lesion sizes of the mutant strains displayed a 25% reduction. The expansion of the lesions was significantly slower in Δ*Bcras2* mutants when compared with that of the wild-type, as observed at 96 h and 120 h (Figure 4B). The Δ*Bcras2* mutants were hardly pathogenic on the green peppers. After 48 h of inoculation, obvious lesions were observed in the wild-type-inoculated pepper wounds, and no lesions were observed in Δ*Bcras2* mutants-inoculated wounds. A small lesion was developed by the Δ*Bcras2-2* mutant after 72 h of inoculation. Until 96 h after inoculation, no lesion was developed on green peppers by the Δ*Bcras2-1* and Δ*Bcras2-3* mutants (Figure 4C,D). Since Δ*Bcras2* mutants were also severely defective in radial growth, it was impossible to conclude whether the reduction in virulence of Δ*Bcras2* mutants was due to defects in pathogenicity or growth. 

### 3.4. Identification of the Potential Downstream Targets of Bcras2 by RNA Sequencing

Compared to the wild-type, Δ*Bcras2* mutants were characterized by reduced growth, altered sensitivity to exogenous stresses, and strongly reduced virulence. To understand the possible downstream targets of *Bcras2* in *B. cinerea*, a comparative RNA sequencing transcriptomics analysis was conducted. RNA was extracted from the wild-type and Δ*Bcras2* mutants, confirmed by nanodrop2000, agarose gel electrophoresis, and 2100 Bioanalyzer, and further used for library construction and sequencing. After filtering the raw data, at least 6.05 Gb clean data for each sample (genome size, 42.65 Mb) was obtained, and the base percentage of Q30 (percentage of bases with sequencing quality above 99.9) was above 94.17%. The alignment rate between the clean data and the reference genome varies from 96.93% to 97.24%. After genome alignment, quality was assessed using the saturation curve, sequencing coverage, and read distribution. To ensure the reliability of biological duplications, correlation analysis was carried out. The correlation coefficient between samples were above 97.2%. 

**Figure 5:**
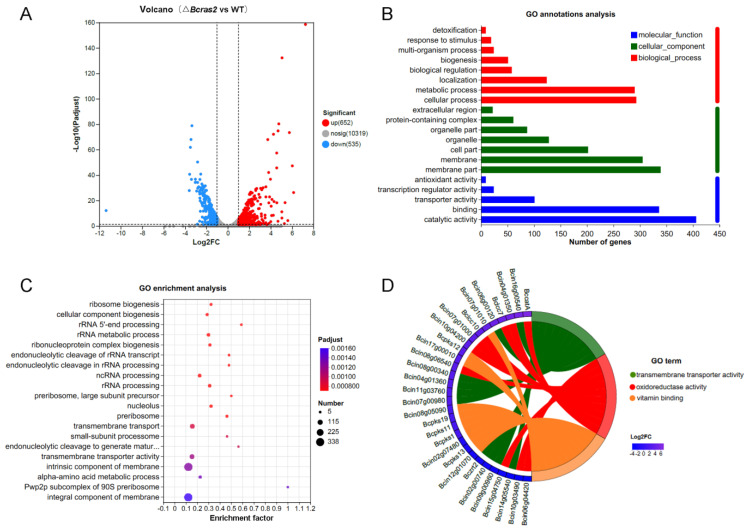
Analysis of differentially expressed genes in Δ*Bcras2* mutants: (**A**) volcano plot of the downstream genes. (**B**) GO annotation analysis of the differentially expressed downstream genes. (**C**) GO enrichment of the genes by Bubble Chart. (**D**) Enrichment Chord for top 10 differentially expressed genes in the first 3 highly enriched GO terms. Benjamini-Hochberg (BH) multiple testing correction was used for the adjusted *p*-values.

A total of 11506 genes were identified. Gene expression level was estimated using TPM. Genes with FC (fold change) more than 2 or less than 0.5 were regarded as significantly differently expressed genes. Approximately 1187 genes were differentially expressed in the Δ*Bcras2* mutant compared to the wild-type, among which 652 genes were up-regulated and 535 genes were down-regulated. To show the distribution of differentially expressed genes more clearly, a volcano plot was constructed with Benjamini-Hochberg (BH) multiple testing correction. The volcano plot showed that the Log2FC (fold change) of most up-regulated genes was concentrated at 1–4, whereas that of the down-regulated genes was at (−1)–(−3). In addition to this, the up-regulated genes were more significant than the down-regulated genes, as shown by the Y-axis of Figure 5A. 

To reveal the molecular functions that the products of differentially expressed genes may perform, the cell components to where they belong, and the biological processes in which they participate, we conducted GO analysis (Figure 5B). For molecular functions, the products of the most differentially expressed genes had binding and catalytic activity. In contrast, other compounds showed transporter, transcription, and antioxidant activities. For cellular components, most gene products were in the membrane part. In addition, genes encoding organelles, protein-containing complexes, and extracellular regions were identified. Most gene products participate in metabolic processes, and other genes are classified as localization, biological regulation, biogenesis, response to stimulus, and detoxification.

To obtain the main functions or metabolic pathways of the differentially expressed genes, we performed GO enrichment analysis using the Goatools software (https://github.com/tanghaibao/Goatools, accessed on 9 September 2022). GO functions with a corrected *p* value (FDR) less than 0.05 was identified to be significantly enriched. Twenty highly enriched GO terms were shown in Figure 5C. The first four most enriched GO terms were integral component of membrane, intrinsic component of the membrane, transmembrane transporter activity, and transmembrane transport, indicating a strong correlation between Bcras2 and the cell membrane. RNA processing and ribosome components items were also detected, such as the pwp2p subcomplex of the 90S preribosome, rRNA processing, ncRNA processing, rRNA metabolic process, rRNA 5′ end processing, and ribosome biogenesis. In addition, the alpha-amino acid metabolic process, small-subunit processome, and nucleolus GO terms were also enriched.

We further revealed the significant differentially expressed genes in the GO terms with high enrichment. The top 10 differentially expressed genes in the first three highly enriched GO terms were selected to draw the enrichment chord. As shown in Figure 5D, the first three highly enriched GO terms were transmembrane transporter activity, oxidoreductase activity, and vitamin binding. Most of the transmembrane transporter genes have not been studied. Oxidoreductase includes Bccat, Bclcc7, Bclcc10, and other unknown genes. Bccat encodes a catalase enzyme that is important for the oxidative stress response. Bclcc7 and Bclcc10 are laccases. Laccases are polyphenol oxidases containing four copper ions. They are extracellular enzymes responsible for virulence and may participate in melanin polymerization in *B. cinerea*. Vitamin-binding proteins include Bcpks1, Bcpks11, Bcpks12, Bcpks13, and Bcpks19. Bcpks13 is responsible for melanin metabolism in conidia, whereas Bcpks12 is responsible for melanin metabolism in sclerotia in *B. cinerea*. These results indicated a strong relationship between Bcras2 and melanin metabolism.

**Figure 6:**
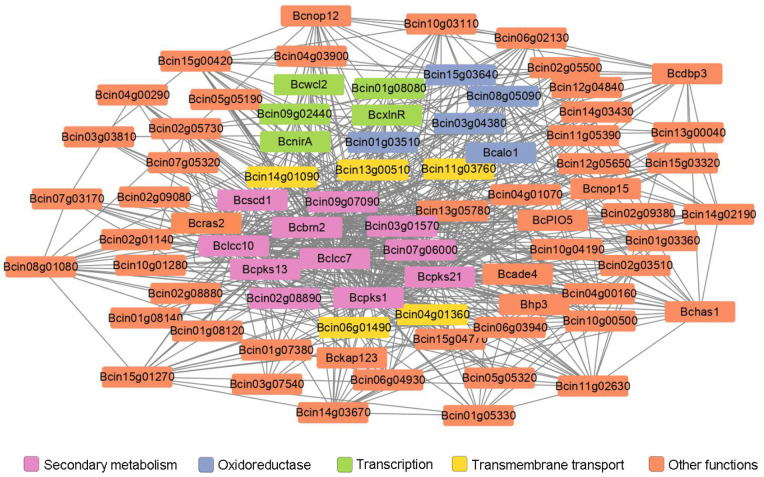
Co-expression analysis of differentially expressed genes in Δ*Bcras2* mutants. Genes highly co-expressed with *Bcras2* were selected. Lines indicate genes co-expressed with a Pearson correlation coefficient of 0.99 or greater; *p*-adjust, 0.05; multiple test correction method: BH.

Co-expression analysis of the differentially expressed genes were performed to reveal gene networks varying in the Δ*Bcras2* mutants. Using the Pearson correlation coefficient, 1804 pairs of genes were identified as co-expressed with a correlation of 0.99 or greater (*p*-value < 0.05). The functions of most co-expressed genes are unknown. Three melanin metabolism genes (*Bcpks13*, *Bcbrn2*, and *Bcscd1*), two laccases (*Bclcc7* and *Bclcc10*) and two polyketide synthases (*Bcpks1* and *Bcpks21*) were found to be co-expressed. Five oxidoreductase (*Bcin08g05090*, *Bcalo1*, *Bcin01g03510*, *Bcin03g04380*, and *Bcin15g03640*) and five transmembrane transport genes (*Bcin11g03760*, *Bcin04g01360*, *Bcin06g01490*, *Bcin14g01090*, and *Bcin13g00510*) were highly co-expressed with melanin metabolism genes. Moreover, five genes (*Bcwcl2*, *BcxlnR*, *BcnirA*, *Bcin09g02440*, and *Bcin01g08080*) participating in transcription were found. Among them, *Bcwcl2* was a blue light receptor responsible for photo-response and sporulation. The co-expressed genes also include two cell wall-related genes (*Bhp3* and *Bcin05g05320*) (Figure 6). *Bhp3* is a hydrophobin-encoding gene responsible for sclerotia development. Although single *Bhp3* deletion mutants has similar phenotypes with the wild-type, double deletion *Bhp1/Bhp3* or triple deletion *Bhp1/Bhp2/Bhp3* mutants have easily wettable sclerotia and apothecia developmental defects [25,26]. 

### 3.5. Validation of the Differentially Expressed Genes by RT-qPCR

**Figure 7:**
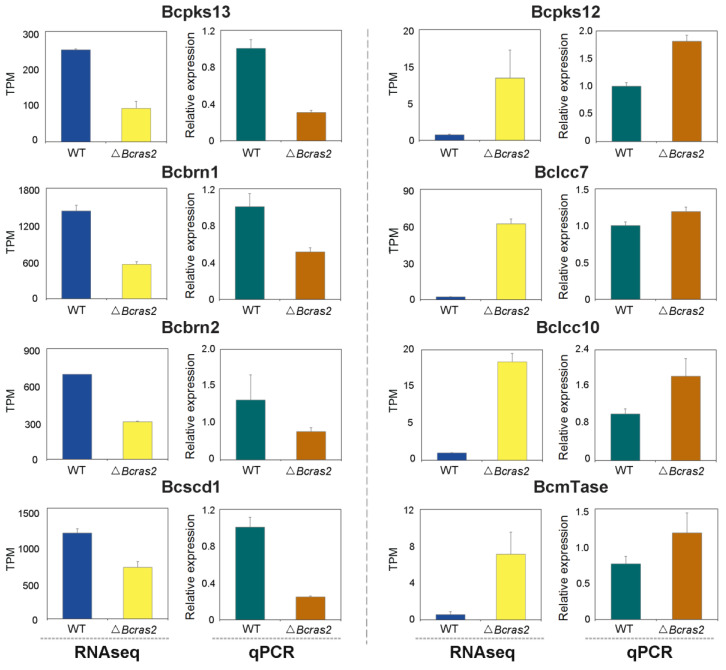
RT-qPCR analysis for differentially expressed genes in RNA sequencing. Bcpks13, Bcpks12: polyketide synthase. Bcbrn1, Bcbrn2: tetrahydroxynaphthalene reductases. Bcscd1: dehydratase. Bclcc7, Bclcc10: laccase. BcmTase: umta methyltransferase.

To validate the differentially expressed genes identified by RNA sequencing, we conducted RT-qPCR experiments. As the differentially expressed genes were closely related to oxidoreductase and melanogenesis regulation, the expression of two oxidoreductase genes, *Bclcc7* and *Bclcc10*, that may participate in melanin polymerization was assessed. Both genes were overexpressed in the Δ*Bcras2* mutants. Five melanogenesis regulation genes, *Bcpks13*, *Bcbrn1*, *Bcbrn2*, *Bcscd1*, and *Bcpks12*, were analyzed. Bcpks13 is a polyketide synthase essential for melanin synthesis in conidia. Bcbrn1 and Bcbrn2 are tetrahydroxynaphthalene reductases with overlapping functions and are responsible for two reduction steps in both the conidia and sclerotia melanin synthesis pathway. Bcscd1 is a dehydratase responsible for two dehydration steps in the melanin synthesis pathway in both reproductive structures. The expression of *Bcpks13*, *Bcbrn1*, *Bcbrn2*, and *Bcscd1* were all repressed in the Δ*Bcras2* mutants compared with that in wild-type, consistent with the results of RNA sequencing. In contrast, *Bcpks12*, which is essential for melanin synthesis in sclerotia, was overexpressed according to both RT-qPCR and RNA-sequencing. Moreover, methyltransferases was reported to regulate the pathogenicity of *B. cinerea* to horticultural crops [27], and the expression of one predicted umta methyltransferase (BcmTase) was selected and detected. Similar to previous genes, umta methyltransferase was overexpressed in the Δ*Bcras2* mutants in both RT-qPCR and RNA sequencing (Figure 7).

### 3.6. The Melanin Metabolic Pathway Is Regulated by Bcras2

The melanin metabolic pathway is essential for pigment formation in grayish conidia and black sclerotia. The pigment in both conidia and sclerotia have been proved to be 1,8-dihydroxynaphthalene (DHN) melanin. However, DHN melanins in different spores are catalyzed by different enzymes. In conidia, DHN melanin is synthesized de novo from acetyl-CoA and malonyl-CoA by Bcpks13, and the generated 2-acetyl-1,3,6,8-tetrahydroxynaphthalene (AT4HN) is deacetylated to 1,3,6,8-tetrahydroxynaphthalene (T4HN) by Bcygh1. In sclerotia, acetyl-CoA and malonyl-CoA are directly converted to T4HN by Bcpks12. The generated T4HN in both conidia and sclerotia is reduced to scytalone by Bcbrn1/2, followed by dehydration to 1,3,8-trihydroxynaphthalene (T3HN) by Bcscd1. T3HN is further reduced to vermelone by Bcbrn1/2. Vermelone is converted into DHN by Bcscd1. 

**Figure 8:**
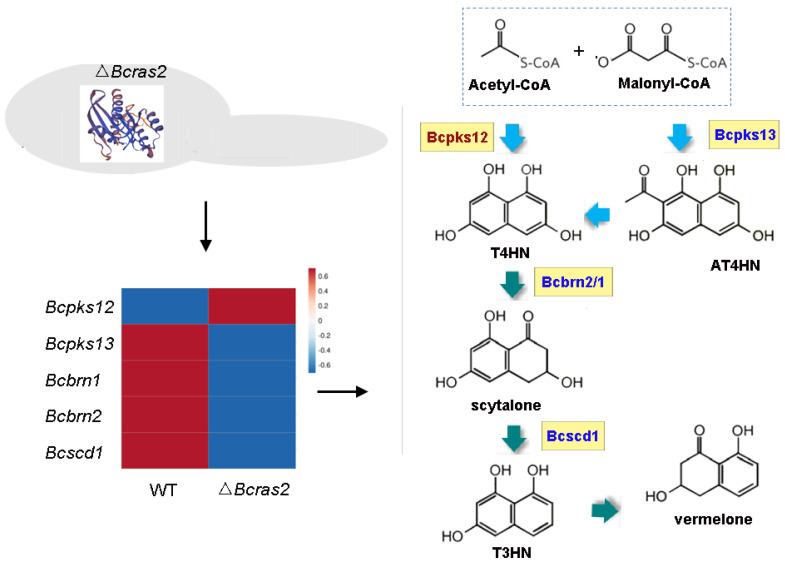
The melanin metabolic pathway is regulated by Bcras2. Bcpks12, Bcpks13: polyketide synthase. Bcbrn1, Bcbrn2: tetrahydroxynaphthalene reductases. Bcscd1: dehydratase. T4HN: 1,3,6,8-tetrahydroxynaphthalene. AT4HN: 2-acetyl-1,3,6,8-tetrahydroxynaphthalene. T3HN: 1,3,8-trihydroxynaphthalene. Right panel, red: up-regulated genes, blue: down-regulated genes.

In the Δ*Bcras2* mutants, Bcpks13, Bcbrn1/2, and Bcscd1 were down-regulated, whereas Bcpks12 was up-regulated (Figure 8). This indicated a reduced pigment in conidia and an enhanced pigment in sclerotia, suggesting an important role of Bcras2 in regulating pigment formation in the reproductive structure of *B. cinerea*. 

## 4. Discussion

Ras GTPases are monomeric G-proteins that serve as signal transduction elements in fungal cells. They transfer signals by toggling between the active guanosine triphosphate (GTP)-binding state and the inactive guanosine diphosphate (GDP)-binding state. Ras proteins have been reported to be essential for growth, development, and virulence [28]. *Fusarium circinatum* is a fungus that causes pine pitch canker disease in many pine species. Deletion mutants of Ras2 in *F. circinatum* produced significantly smaller colonies, delayed conidial germination, and reduced virulence [29]. In *B. cinerea*, Δ*Bcras2* mutants showed more sclerotia production besides reduced growth and virulence (Figure 2 and Figure 4). Sclerotia are reproductive structures generated by *B. cinerea* [30]. The transformation of conidia and sclerotia is often related to environmental factors [31]. When environmental conditions are appropriate, *B. cinerea* produces asexual conidia, which are light and are conducive to spreading. When the environment becomes adverse, sclerotia are formed [32]. The sclerotia have thick epidermis to protect the cell from moisture and nutrients loss and help the cell to survive harsh environments [26]. The increased sclerotia production in the Δ*Bcras2* mutants indicates a close relationship between *Bcras2* and environmental responses. 

Environmental conditions play an important role in the invasion of *B. cinerea*. Under humid and low-temperature conditions, *B. cinerea* is more likely to infect host plants, causing gray mold disease in plants and causing economic losses to humans [33]. However, on humid nights and sunny days, *B. cinerea* may cause noble rot in overripe grapes, which is beneficial to humans [34]. Many studies have shown that Ras proteins are related to environmental responses. *Candida albicans* is a human commensal fungal pathogen that causes opportunistic infections. It has yeast and hyphal forms, and its morphogenesis is critical for its virulence [35]. Ras signalling was reported to be crucial for the integration of environmental cues with morphogenesis [36]. Strains with the dominant active Ras1(V13) allele are inclined to form hyphae, whereas strains carrying the dominant negative Ras1(A16) allele show reduced hyphal growth [37]. *Paracoccidioides brasiliensis* is a dimorphic fungus that can survive host nitrosative stress and cause paracoccidioidomycosis (PCM) in human phagocytic cells. The Ras-GTPase and Hog1 MAPK pathways contribute to nitrosative stress by regulating the expression of antioxidant genes [38]. In *B. cinerea*, *Bcras2* is closely related to oxidative stress and cell wall stress. Mutant strains lacking *Bcras2* were more sensitive to H_2_O_2_ stress than the wild-type strains. However, the *Bcras2* deletion strains were resistant to cell wall stress and grow better with Congo red (150 and 300 µg mL^−1^) or SDS (0.005%). Interestingly, when the concentration of SDS was increased to 0.01%, the mutant was still less sensitive than the wild-type, but its growth was inhibited (Figure 3). Thus, Ras2 was responsible for the environmental response in *B. cinerea*, positively regulating H_2_O_2_ stress and negatively regulating stress response to Congo red and SDS. The cell membrane is a functional link between the external environment and regulation of inner gene [39]. The membrane is essential for the function of Ras proteins and their downstream signaling [40]. Multiple differentially expressed genes related to the cell membrane were identified in the Δ*Bcras2* mutants (Figure 5). Considering that the lack of *Bcras2* is conducive to the formation of sclerotia with dense structure (Figure 2), it is speculated that after *Bcras2* deletion, the expression of cell membrane protein changes, the cell wall and cell membrane become dense, and *B. cinerea* transfers from the rapid growth stage to the stress tolerance stage. Cell wall destructors promote growth by reducing the density of cell wall. When the concentration of SDS is increased to 0.01%, its damage to the cell wall is greater than the promotion of growth, so the growth was inhibited.

*B. cinerea* generates grayish conidia for dissemination or black sclerotia for survival in adverse environments. The pigment responsible for the longevity of the conidia and sclerotia has been demonstrated to be the 1,8-dihydroxynaphthalene (DHN) melanin [41,42]. DHN synthesis is initiated by acetyl coenzyme A (acetyl-CoA) and malonyl coenzyme A (malonyl-CoA), catalytic substrates of polyketide synthase (PKS) [43]. In *B. cinerea*, there are two developmentally regulated PKS genes, *Bcpks12* and *Bcpks13* [41]. *Bcpks12* is responsible for sclerotia development and generates T4HN for subsequent DHN synthesis [44]. *Bcpks13* is expressed during conidia development and produces AT4HN, which can be further converted to T4HN by hydrolases Bcygh1 [41,45]. The path downstream of Bcpks is shared by both reproductive spores. T4HN is alternately dehydrated and reduced twice to form DHN. Dehydration is catalyzed by Bcbrn1/Bcbrn2 [41], and the reduction is catalyzed by Bcscd1 [32]. The subcellular distribution of Bcpks12, Bcpks13, Bcygh1, Bcbrn1/Bcbrn2, and Bcscd1 are delicately orchestrated, perhaps to ensure enzymatic efficiency and protect them from the toxic intermediate metabolite [42]. After DHN monomers are synthesized, they are speculated to form polymers by laccases [41]. The melanin regulatory pathway is regulated by transcription factors and light [46,47]. Bcpks12 is positively affected by the physically linked transcription factor Bcsmr1, whereas Bcpks13 is promoted by its physically linked transcription factor Bcztf1 and Bcztf2 [41]. The expression of *Bcztf1* and *Bcztf2* was enhanced, whereas that of *Bcsmr1* was repressed by a light-responsive transcription factor (Bcltf1) [48]. In our study, *Bcpks12* was overexpressed, whereas *Bcpks13*, *Bcbrn1*, *Bcbrn2*, and *Bcscd1* were repressed (Figure 7). Therefore, the absence of *Bcras2* contributes to the formation of melanin in sclerotia but is not conducive to the formation of melanin in conidia. The transcription factor *Bcsmr1* was up-regulated in transcriptome studies, however, its expression was inconsistent in different batches of RT-qPCR experiments. *Bcsmr1* was reported to be differentially expressed in the life cycle [41], perhaps because of subtle differences in culture conditions and growth status. Thirteen laccases have been found in *B. cinerea*. Among them, *Bclcc4*, *Bclcc5*, *Bclcc8*, and *Bclcc9* were overexpressed in the hyper-sporulating mutants Δ*Bcltf1* in a similar manner to *Bcpks13*, *Bcygh1*, *Bcbrn1*, *Bcbrn2*, and *Bcscd1* [41,48], indicating that they were closely related to melanin polymerization in conidia. In this study, *Bclcc4* was slightly overexpressed in Δ*Bcras2* mutants, *Bclcc5* was repressed, and *Bclcc8* and *Bclcc9* showed similar expression in Δ*Bcras2* mutants and the wild-type. However, two other laccases (*Bclcc*7 and *Bclcc*10) were significantly overexpressed in Δ*bcras2* mutants. Considering that the reproductive spores of *B. cinerea* have different melanin biosynthesis pathways, it is speculated that the enzymes used for pigment polymerization in the conidia and sclerotia are different. *Bclcc*7 and *Bclcc*10 may be responsible for the pigment polymerization of DHN in sclerotia. 

Light has a significant regulatory effect on the reproductive structures of *B. cinerea*. *B. cinerea* tends to produce conidia under light conditions and sclerotia in the dark [49]. In our experiment, sclerotia production increased and conidia production decreased in the Δ*bcras2* deletion mutants (Figure 2), indicating that Bcras2 negatively regulates sclerotia production. The relationship between Bcras2 and light exposure requires further investigation. In addition, Bcras2 was speculated to activate the adenylate cyclase and the Gα subunits Bcg1 and Bcg3 in *B. cinerea* because exogenous cAMP partially restored the germination and growth defects of Δ*bcras2* mutants [17]. However, adenylate cyclase (Bac, enzymes for cAMP synthesis) and Gα subunits showed similar expression in Δ*bcras2* mutants and the wild-type in the RNA sequencing experiment. Furthermore, the expression of phosphodiesterase (*Bcpde1* and *Bcpde2*) responsible for cAMP degradation was not affected in the Δ*bcras2* deletion mutants, indicating that *Bcras2* did not affect the expression of *Bac*, *Bcpde1*, and *Bcpde2* at the transcriptional level. Thus, the relationship between Bcras2 and the cAMP signaling pathway requires further study. 

## 5. Conclusions

In this study, the function of the Bcras2 protein in *B. cinerea* was analyzed by gene deletion and RNA sequencing transcriptomics. It was found that Bcras2 was important for regulating growth, sclerotia production, virulence, stress response, and melanin-related gene expression. However, the relationship between Bcras2 and light requires further study, since the phenotypes and melanin-related genes expression of Δ*Bcras2* partially overlapped with those regulated by light. In addition, the role of Bcras2 in the cAMP signaling pathway was not found by RNA sequencing and should be further verified. The results of this study will provide an impetus for the further reveal of the relationship between Bcras2 and the light response in *B. cinerea*, as well as its role in the cAMP signal pathway. 

## Data Availability

The data presented in this study are openly available in NCBI (https://www.ncbi.nlm.nih.gov/bioproject/PRJNA941123, uploaded on 6 March 2023).

## Supplementary Materials

The following supporting information can be downloaded at: https://www.mdpi.com/article/10.3390/jof9040432/s1, Table S1: Primers used in this experiment.
