# Supplementary material for: Ras2 Is Responsible for the Environmental Responses, Melanin Metabolism, and Virulence of Botrytis cinerea
Source: J Fungi (Basel). 2023 Mar 31;9(4):432. doi: 10.3390/jof9040432 (PMC10142356; doi:10.3390/jof9040432)
Supplement: Supplementary file 1 [file jof-09-00432-s001.zip › jof-2234244-supplementary.pdf]

Table S1. Primers used in this experiment.

No.	Primer	Sequence (5'-3')
1	*bcras2*-L-up	CGTTGTAAAACGACGGCCAGTGCCAGTCCAACACGAACCACCAACT
2	*bcras2*-L-down	ATGCATGGTTGCCTAACTCGGCGCGCTTTGAAGACGGAGCTTTGGC
3	*bcras2*-R-up	CTAGAGGATCCCCGGGTACCGAGCTGAAATTCATCTCCTTACACTTCAAGAT
4	*bcras2*-R-down	ACAGCTATGACCATGATTACGAATTGCTGTCCATTCCATCATTCCC
5	HPH1	CAGCTTCGATGTAGGAGG
6	HPH2	ACTTCGGGGCAGTCCTCG
7	southern-up	CTCACCCCCATCTCAACTCC
8	southern-down	CTCCATACAAGCCAACCACG
9	UCE-up	ATCACCCAAACATCAACT
10	UCE-down	CATAGAGCAGATGGACAA
11	*bcpks*12-up	CCTCAGCAACAGCCTCAGTG
12	*bcpks12*-down	CGAACGCATAGGTGGGTAAA
13	*bcpks13*-up	GTTGAAATTGGCCCACACCC
14	*bcpks13*-down	GCGTCGGCATTTCTGCTTAG
15	*bcbrn1*-up	AGATGATGCTGGGTTTGGGG
16	*bcbrn1*-down	TTCGGGGTCGAATGAACCAG
17	*bcbrn2*-up	ACCATGCTTTGTACGCTGGA
18	*bcbrn2*-down	CATGCCTTCAAATCCGCCTG
19	*bcscd1*-up	CCCAACATCTTCTCGGTGCT
20	*bcscd1*-down	GGCATGACTGTGACCCTTCA
21	*bclcc7*-up	GTGAAGGAATGCTCGGTGGA
22	*bclcc7*-down	GTCCAGCTTGAGAATGGGCT

23	*bclcc10*-up	ATCAATTTCCCGGACCGCTT
24	*bclcc10*-down	GCCAATGAAGACTCAGCCCT
25	*bcmTase*-up	TCCCAAGGGTGTTTATGCCC
26	*bcmTase*-down	GAAATCTCGCGTGCCACATC
